# News and Coming Events

**Published:** 1909-04-10

**Authors:** 


### News and Coming Events.

Viscount Clifden, Deputy Chairman of the Appeal Committee of the Mount Vernon Hospital for Consumption and Diseases of the Chest, will preside at a festival dinner at the Hôtel Cecil on Thursday, May 13, in aid of this hospital's appeal for £10,000 to carry on its work.

The Duke of Devonshire has been unanimously elected President of the West End Hospital for Nervous Diseases, in succession to the late Lord Egerton of Tatton, and Mr. H. C. Willock-Pollen has been elected Vice-Chairman in the place of the late Mr. A. W. Elam.

At the annual graduation ceremonial at the University of St. Andrews, which took place on March 31, the honorary degree of LL.D. was conferred upon Dr. James Wallace, D.Sc., M.D., F.R.S., Processor of Chemistry in the University of Edinburgh.

It was officially stated in Paris on April 2 that there were then ninety-two cases of cerebro-spinal meningitis in that city and its suburbs. The patients are being treated with the serum associated with the name of Dr. Wasserman of the Koch Institute in Berlin, which has for some time been successfully used in the French military hospitals.

The Radcliffe prize for 1909 in the University of Oxford has been awarded by the Master and Fellows of University College, upon the report of the examiners, to Dr. Arthur Frederick Hertz, formerly Demy of Magdalen College. The subject of Dr. Hertz's dissertation was "The Physiology and Pathology of the Movement of the Intestines."

Mr. Charles Ernest Baker, M.B., F.R.C.S. Eng., died suddenly last week at the age of forty-four at his residence in South Kensington. At an inquest the cause of death was stated to be heart failure from an overdose of veronal, and a verdict of death by misadventure was returned. Mr. Baker obtained a first class in the Natural Science Tripos at Cambridge, and in 1886 the M.B. and B.C. degrees, and he obtained the F.R.C.S. in 1893. He had been a house surgeon at St. Bartholomew's Hospital and at the East London Hospital for Children, and a resident medical officer at the Royal Free Hospital.

The Duke of Abercorn, accompanied by the Duchess, presided at the annual meeting of the West London Hospital on March 31. Among those present were the Bishop of London, the Mayor of Hammersmith, and the High Master of St. Paul's School. The annual report stated that the in-patients in 1908 numbered 2,273 and the outpatients 38,297. The Chairman announced that Lady Bergne had promised to endow a cot with £525. He expressed the great regret shared by all at the death of Lord Glenesk, who was a vice-president and chairman of the hospital.

Sir William Allchin was on March 31 presented with his portrait in oils at Westminster Hospital. The presentation was made by Sir John Wolfe Barry, chairman of the House Committee of the hospital, who referred to the service which Sir William Allchin had rendered to the medical profession in general and to the Westminster Hospital in particular. The portrait is the work of Sir Luke Fildes, and is a fitting souvenir of the high respect and affection which Sir William has so long inspired amongst all sections of those in any way connected with the Westminster Hospital.

Dr. Squire Sprigge, who has been chief of the editorial staff of *The Lancet* for the past fifteen years, has been formally appointed editor of that paper, in succession to the late Mr. Thomas Wakley.

Princess Christian visited Purley on March 31 in order to open a Cottage Hospital built by subscriptions raised in the district. The High Sheriff of Surrey (Sir Frederick T. Edridge) welcomed her Royal Highness on behalf of the committee. He pointed out that provision had been made for future additions to the building, and that the sum still needed to meet the cost was £500. After a short service, conducted by the Vicar of Purley, purses were received by Princess Christian, and her Royal Highness opened the hospital with a key presented by the hon. architect, Mr. J. Newton.

The Lady Mayoress presided on the afternoon of March 31 at a drawing-room meeting at the Mansion House in connection with the Royal Institute of Public Health, for the purpose of calling attention to the need of hygiene in the home. It was announced that Her Majesty the Queen and Her Royal Highness the Princess of Wales had consented to become patroness and vice-patroness respectively of the Ladies' Committee in course of formation. Professor W. R. Smith, President of the Institute, having explained the scope of its work, Sir John Cockburn moved, and Professor Sims Woodhead seconded, a resolution in which the meeting recorded its approval of the action of the Institute in promoting classes in domestic economy for the instruction of those visiting the homes of the poor.

At the London Hospital on April 1, Mr. Wynne Baxter held an inquest on the body of Mr. Angus Bewley Wilson, M.B. Cantab., whose death occurred there on Wednesday last. Mr. Wilson was attending a woman who was suffering from suicidal laudanum poisoning, and in her struggles she bit his finger. Blood poisoning ensued, and death occurred within a week of the injury. Mr. Wilson was thirty-two years of age and a house physician at the London Hospital. The Coroner said that Mr. Wilson apparently did not pay so much attention to his own wound as he would have done to the wounds of other people. It was another life sacrificed in the interests of other people. There was no doubt that there was a great deal of generous conduct and self-sacrifice on the part of doctors. The jury returned a verdict of death by misadventure.

At the Kingston Police Court on April 1, a gentleman was summoned by the police for exceeding the speed limit at Cobham on March 21. A police sergeant said he timed the car at 29 miles an hour. When it was stopped an occupant of the car said he was Mr. Stansfield Collier, and was in a hurry to reach London to perform an operation. The witness replied that only three weeks ago Mr. Collier had told him the same tale, and that it would not do again. A house surgeon at St. Mary's Hospital stated that on the evening in question he had a critical case and telephoned to Dr. Collier, who was in Surrey, to come at once, and the latter arrived at the hospital at 10.20. Dr. Collier said he gave instructions to the driver of the car, which he borrowed because it was faster than his own, to drive as quickly as possible with due regard to safety. If he had not been stopped by the police he believed he would have saved the man's life. The Bench, under the special circumstances, thought it inexpedient to impose a penalty, and dismissed the summons, but they held that the police were justified in prosecuting.

